# Protective behavior or ‘true’ tool use? Scrutinizing the tool use behavior of ants

**DOI:** 10.1002/ece3.6968

**Published:** 2020-11-02

**Authors:** Gábor Módra, István Maák, Ádám Lőrincz, Orsolya Juhász, Péter János Kiss, Gábor Lőrinczi

**Affiliations:** ^1^ Department of Ecology University of Szeged Szeged Hungary; ^2^ Doctoral School of Environmental Sciences University of Szeged Szeged Hungary; ^3^ Museum and Institute of Zoology Polish Academy of Sciences Warsaw Poland; ^4^ Doctoral School in Biology Faculty of Science and Informatics University of Szeged Szeged Hungary

**Keywords:** *Aphaenogaster subterranea*, debris dropping, defense response, food transport, foraging tool use

## Abstract

In the genus *Aphaenogaster,* workers use tools to transport liquid food to the colony. During this behavior, ants place or drop various kinds of debris into liquids or soft food, and then, they carry the food‐soaked tools back to the nest. According to some authors, this behavior is not "true" tool use because it represents two separate processes: a defense response to cover the dangerous liquid and a transport of food. Here, we investigated the debris dropping and retrieving behavior of the ant *Aphaenogaster subterranea* to establish which of the two hypotheses is more probable by conducting manipulative experiments. We tested the responses of eight colonies (a) to liquid food (honey‐water) and nonfood liquids (water) in different distances from the nest and (b) to nonthreatening liquids previously covered or presented as small droplets. We also tested whether the nutritional condition of colonies (i.e., starved or satiated) would affect the intensity and rate of debris dropping. Our results were consistent with the tool‐using behavior hypothesis. Firstly, ants clearly differentiated between honey‐water and water, and they directed more of their foraging effort toward liquids farther from the nest. Secondly, ants performed object dropping even into liquids that did not pose the danger of drowning or becoming entangled. Lastly, the nutritional condition of colonies had a significant effect on the intensity and rate of object dropping, but in the opposite direction than we expected. Our results suggest that the foraging behavior of *A. subterranea* is more complex than that predicted by the two‐component behavior hypothesis and deserves to be considered as "true" tool use.

## Graphical abstract

In the genus Aphaenogaster, workers use various materials to transport liquid food to the colony. According to some authors, this behavior represents two separate processes: a defense response to cover the dangerous liquid and a transport of food. Our results with *Aphaenogaster subterranea*suggest that this behavior is more complex than that predicted by the two‐component behavior hypothesis and deserves to be considered as "true" tool use.

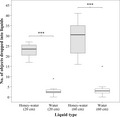

## 1 INTRODUCTION

Tool use is “...the exertion of control over a freely manipulable external object (the tool) with the goal of (a) altering the physical properties of another object, substance, surface or medium (the target, which may be the tool user or another organism) via a dynamic mechanical interaction, or (b) mediating the flow of information between the tool user and the environment or other organisms in the environment” (St. Amant & Horton, 2008). From the ten categories of tool use (food preparation, food extraction, food transport, food capture, physical maintenance, mate attraction, nest construction, predator defense, agonism, other), four are associated with some kind of food manipulation (Bentley‐Condit & Smith, 2010). Food transport, that is, the use of tools to transport food and/or water, is a well‐documented phenomenon in some vertebrates (birds and primates), but among nonvertebrate animals is known only in a few ant species including members of the genus *Aphaenogaster* (Bentley‐Condit & Smith, 2010; Shumaker et al., 2011). During this behavior, ants place or drop various debris (e.g., pieces of leaf, soil, and wood) into or over soft or liquid foods, and after an interval, they retrieve and carry the food‐soaked tools back to the nest where nestmates can feed on them (e.g., Banschbach et al., 2006; Fellers & Fellers, 1976). This is a highly efficient way of transporting food, since, by using various objects as tools, foraging workers are capable of transporting much larger amounts of liquids than they could transport internally in their nondistensible crop (Fellers & Fellers, 1976; Tanaka & Ono, 1978). By allowing efficient transportation and sharing of food, tool use may also represent a compensation for the absence of oral trophallaxis (i.e., the exchange of liquid food stored in the crop by regurgitation) in some tool user species, such as members of the genera *Aphaenogaster*, *Messor,* and *Pogonomyrmex* (Agbogba, 1985). From the 205 described species of the genus *Aphaenogaster* (Bolton, 2020), at least 10 are known to use tools for food transport (Agbogba, 1985; Banschbach et al., 2006; Cerdá et al., 1988; Fellers & Fellers, 1976; Fowler, 1982; Lőrinczi, 2014; Lőrinczi et al., 2018; Maák et al., 2017; McDonald, 1984; Módra et al., 2017; Tanaka & Ono, 1978). Similar behavior has been observed in a few other myrmicine species such as *Messor barbarus* (Durán, 2011), *Messor structor* (Módra et al., 2017), *Novomessor albisetosus* (McDonald, 1984; Wetterer et al., 2002), *Pogonomyrmex badius* (Morrill, 1972), and *Solenopsis invicta* (Barber et al., 1989; Qin et al., 2019).

Despite the growing number of publications in this area, some authors still question the idea that the debris dropping and retrieving behavior of ants constitute "true" tool use. Even Hölldobler and Wilson (1990) criticized this view in their classical book and claimed that debris dropping functions merely to cover liquids in the nest vicinity, thereby protecting other workers from drowning or becoming entangled. Indeed, a long‐standing observation is that many ant species show the tendency to cover, and in some cases entirely bury unmoveable, disagreeable substances with debris as a protective behavior (Wheeler, 1910). Durán (2011) who studied the foraging behavior of the harvester ant *M. barbarus* on honey baits also reached a similar conclusion. Durán (2011) suggested that the debris dropping and retrieving behavior of ants involve, in fact, two separate processes. The first part of this two‐component behavior is the dropping of debris into honey as a general defense response against drowning or entanglement, whereas the second part, the retrieving of debris imbued with honey is simply a transport of food. According to the author, the significant time lag between food cover and transport is an indicative of the independence of the two behavioral components, just as the fact that due to this time delay, different workers perform debris dropping and retrieving. The author also refers to the observations of Agbogba (1985), who noted that honey mixed with soil particles failed to stimulate the dropping behavior in *Aphaenogaster subterranea* and *Aphaenogaster senilis* but triggered the retrieving behavior of workers.

Other studies, on the other hand, suggest that the debris dropping and retrieving behavior of ants are more complex than the aforementioned hypotheses imply. For instance, McDonald (1984) showed that beyond a certain distance from the nest, the workers of *Novomessor albisetosus* were selective to different liquids offered them as baits. As these were placed farther from the nest, ants reduced or stopped dropping soil particles into water but continued doing so into honey‐water, indicating that soil dropping is not just a general defensive response to any liquid. Other studies also show that the debris dropping behavior is selective when it comes to nonfood liquids. It occurs in a sporadic manner in some cases (Lőrinczi, 2014; Lőrinczi et al., 2018; Maák et al., 2017; McDonald, 1984), while in others nonfood liquids are ignored even in the close vicinity of the nest (Agbogba, 1985; Banschbach et al., 2006). In the study of Banschbach et al. (2006), the debris dropping and retrieving activity of the workers of *Aphaenogaster rudis* were higher at liquid baits placed closer to the nest, though they ceased it after a period of time, probably because the colony became satiated. After a few days, however, ants restarted debris dropping and retrieving at baits. Their foraging behavior thus corresponded to the food demand of the colony, rather than continuing until all food was covered, as it would be expected if debris dropping represented a general defense response against drowning or entanglement. Individual marking was also carried out in this study, and it was found that the tool‐using behavior was carried out by a small subset of foraging workers; that is, individuals involved in debris dropping were the same ones that retrieved the food‐soaked debris.

How should we then interpret the debris dropping and retrieving behavior of ants? Does it represent a two‐component behavior involving two separate processes (i.e., defense response and food transport) or does it constitute "true" tool use? Each of these hypotheses provides testable predictions, which can be readily tested through manipulative experiments. If debris dropping represents only a general defense response against drowning or entanglement (H1), we can predict that (a) there would be no difference in the number of objects dropped into liquid food and nonfood liquids, (b) more objects would be dropped into liquids closer to the nest than into those farther from the nest, (c) no objects would be dropped into liquids previously covered with debris, (d) no objects would be dropped into liquids presented as small droplets, and (e) the nutritional condition of colonies (i.e., starved or satiated) would not affect the intensity and rate the dropping of objects. However, if the purpose of debris dropping is to promote food transport (H2), we can predict that (a) more objects would be dropped into liquid food than into nonfood liquids, (b) more objects would be dropped into liquids farther from the nest than into those closer to the nest, (c) objects would be dropped into liquids previously covered with debris, especially if these objects are more manageable, (d) objects would be dropped into liquids presented as small droplets, and (e) the nutritional condition of colonies (i.e., starved or satiated) would have an effect on the intensity and rate of the dropping of objects.

## 2 METHODS

### 2.1 Study species

Our study species, *Aphaenogaster subterranea* (Latreille, 1798), is distributed in Southern and Central Europe, Moldova, Crimea, the Caucasus, Asia Minor, and the Near East (Czechowski et al., 2012). Its typical habitats are warm and moderately humid deciduous forests, but it is also found in pine forests, in hedgerows, and among shrubs in dry grasslands (Czechowski et al., 2012; Lőrinczi, 2011; Seifert, 2018). Nests are usually built in the soil, generally under stones or at bases of trees, sometimes in decaying wood or in leaf litter (Czechowski et al., 2012; Lőrinczi, 2011; Seifert, 2018). Colonies are probably monogynous with up to several thousand individuals (Czechowski et al., 2012; Seifert, 2018).

### 2.2 Laboratory experiments

We worked with eight colonies of *A. subterranea*, each with approximately 500–600 workers, queens, and brood of all stages, which were collected from a mixed black pine (*Pinus nigra*) forest near the village of Litér (West‐Hungary). Colonies were housed in plastic boxes (L 28 cm × W 20 cm × H 12 cm) provided with nesting materials (soil and plant fragments) from the ants’ natural habitat. Each nest box was connected to a foraging arena (L 60 cm × W 30 cm × H 15 cm) with a plastic tube (1 cm in diameter, 10 cm in length). We kept the colonies under constant conditions (temperature 24 ± 4°C; relative humidity 42%–43%; 12‐hr L:D cycle). Water was always available ad libitum, while food was offered every second day, except before the experiments to avoid ants being too satiated. Colonies were fed with a commonly used artificial diet (Bhatkar & Whitcomb, 1970).

We designed four experiments (Exp. 1, Exp. 2, Exp. 3, and Exp. 4) to test forager responses to different situations (Table 1). During the experiments, we observed the behavior of each of the eight colonies for 1‐min and repeated after every 4‐min for three consecutive hours (from 8:30 a.m. to 11:30 a.m.). The experiments were conducted in different trials: in Exp. 1 the distance, in Exp. 2 and Exp. 3 the type of liquid was changed, with 2 days of break between the trials. In Exp. 4, the nutrition condition of colonies was changed every 2 weeks (altogether for 6 weeks).

**Table 1:** Experimental designs used in the study

	Experiment 1		Experiment 2		Experiment 3		Experiment 4		
	Trial 1	Trial 2	Trial 1	Trial 2	Trial 1	Trial 2	Trial 1	Trial 2	Trial 3
Liquid type	Honey‐water and water	Honey‐water and water	Honey‐water covered with pine needles	Water covered with pine needles	Honey‐water	Water	Honey‐water	Honey‐water	Honey‐water
Liquid setting	One single drop, respectively	One single drop, respectively	One single drop	One single drop	10 small droplets	10 small droplets	One single drop	One single drop	One single drop
Liquid distance from nest entrance	20 cm	60 cm	40 cm	40 cm	40 cm	40 cm	40 cm	40 cm	40 cm
Objects as potential tools	All 5 objects	All 5 objects	All 5 objects	All 5 objects	All 5 objects	All 5 objects	Pine needles	Pine needles	Pine needles
Nutritional condition of colonies	Normal	Normal	Normal	Normal	Normal	Normal	Normal	Starved	Satiated

Two liquids were used in the experiments, water and honey‐water (1:3), from which the latter was used as a representative liquid food source. The liquids were placed on 4‐cm‐diameter plastic disks, which were then placed into the foraging arenas. The experiments tested forager responses to variations in the size and accessibility of liquid droplets and their distance from the nest entrance. The following objects as potential tools were used during the experiments: small soil grains (ca. 1 mm in diameter), large soil grains (ca. 2 mm in diameter), pieces of pine needles (ca. 8 mm in length), pieces of leaves (ca. 3 mm in diameter), and pieces of sponges (ca. 3 mm in diameter).

In three experiments (Exps. 1, 2, and 4), the liquids used consisted of a single drop (ca. 0.5 ml and 2 cm in diameter) placed in the center of the disk. In Exp. 1, one disk with water and one disk with honey‐water were simultaneously placed in the foraging arena. In Exp. 2 Trial 1, a honey‐water drop and in Trial 2 a water drop was used, and the liquids were fully covered with 10–12 pieces of pine needles. In Exp. 3 Trial 1, honey‐water and in Trial 2 water was presented in 10 randomly dispersed small droplets (ca. 25 × 10^–5^ ml and 1 mm in diameter), respectively. Water droplets were supplemented in every 5 min because of evaporation. In Exp. 4, only honey‐water was used in 3 trials: Trial 1 was performed under the standard nutrition of colonies (feeding every second day), Trial 2 after a 2‐week starvation period, and Trial 3 after a 2‐week satiation period, where food was available ad libitum to the ants.

Regarding the distance from the nest, in Exp. 1 Trial 1, disks were placed 20 cm from the nest entrance and in Trial 2 the setting was repeated with the disks placed 60 cm from the nest entrance. In all other experiments (Exps. 2, 3, and 4), the liquids were placed 40 cm from the nest entrance in all trials.

In Exps. 1, 2, and 3, we provided all the objects for the ants (small soil grains, large soil grains, pieces of pine needles, pieces of leaves, and pieces of sponges). These were mixed in equal volume, and a mixture of ca. 7 cm^3^ was piled up 4 cm from the liquids to avoid the effect of distance during the object selection of ants (see Lőrinczi et al., 2018). In all 3 trials of Exp. 4, we provided only 30 pieces of pine needles for the ants, placed 4 cm from the disk in a countable manner.

During the observations, the following data were recorded: the number of workers dropping objects into liquids; the number of workers carrying food‐soaked objects from the liquids back to the nest; the number and type of objects dropped into liquids; and the number and type of food‐soaked objects carried back to the nest. The fourth experiment was an exception, where we recorded only the number of pine needles remaining in the object piles. With this setting, we were able to easily count the objects and precisely follow the intensity of their dropping into honey‐water in spite of the short (1‐min) observation periods. However, neither the number of objects dropped into honey‐water nor the number of retrieved objects could be determined due to the simultaneous dropping and retrieving activity of the ants.

### 2.3 Data analysis

In the first setup, the effect of the liquid type and distance on the summed number of objects dropped into liquids was analyzed with the help of generalized linear mixed models (GLMMs, negative binomial error term, maximum‐likelihood fit). In the full model, the liquid type and their distance from the nests were included as explanatory factors. We analyzed in separate models the effect of object type and liquid type on the number of objects dropped into liquids at the two different distances (GLMMs, negative binomial error term, maximum‐likelihood fit). In the full models, the object type, the liquid type, and their interaction were included as explanatory factors. We used the same model constructions to analyze the effect of different variables on the summed number of objects, and the object type‐dependent number of objects retrieved from liquids at the two distances.

In the second and third setups, the effect of the liquid type on the summed number of objects dropped into liquids was also analyzed with the help of GLMMs (negative binomial error term, maximum‐likelihood fit). In the full model, the liquid type was included as an explanatory factor. We analyzed in separate models the number of objects dropped into the two different liquid types (GLMMs, negative binomial error term, maximum‐likelihood fit). In the models, the object type was included as an explanatory factor. We used the same model constructions to analyze the effect of different variables on the summed number of objects, and the number of objects depending on the object type retrieved from liquids.

In the fourth setup, the dropping rate of pine needles on the liquids was analyzed with Cox regression, whereas the difference in the number of objects dropped into liquids by the colonies with a different nutritional state was analyzed with GLMMs (binomial error term, maximum‐likelihood fit). In our models, the nutritional state of colonies was included as an explanatory factor. The removal of objects from the liquids could not be determined precisely due to the experimental setup, so the data were not analyzed.

In every model, the colony ID was included as a random factor. The best models were determined with automated model selection. All statistical analyses were carried out in the R Statistical Environment (R Core Team, 2016). GLMMs were performed using *glmer.nb* function in lme4 package (Bates et al., 2013), automated model selection with the help of *dredge* function in MuMIn package (Bartoń, 2013), whereas Cox regression analysis with the help of *coxme*function in Coxme package (Therneau, 2015). Post hoc sequential comparisons (Tukey HSD) among factor levels when performing GLMM and Cox regression analysis were performed using *lsmeans* function from lsmeans package (Russell, 2016).

## 3 RESULTS

### 3.1 Experiment 1: liquid choice and distance

**Figure 1:**
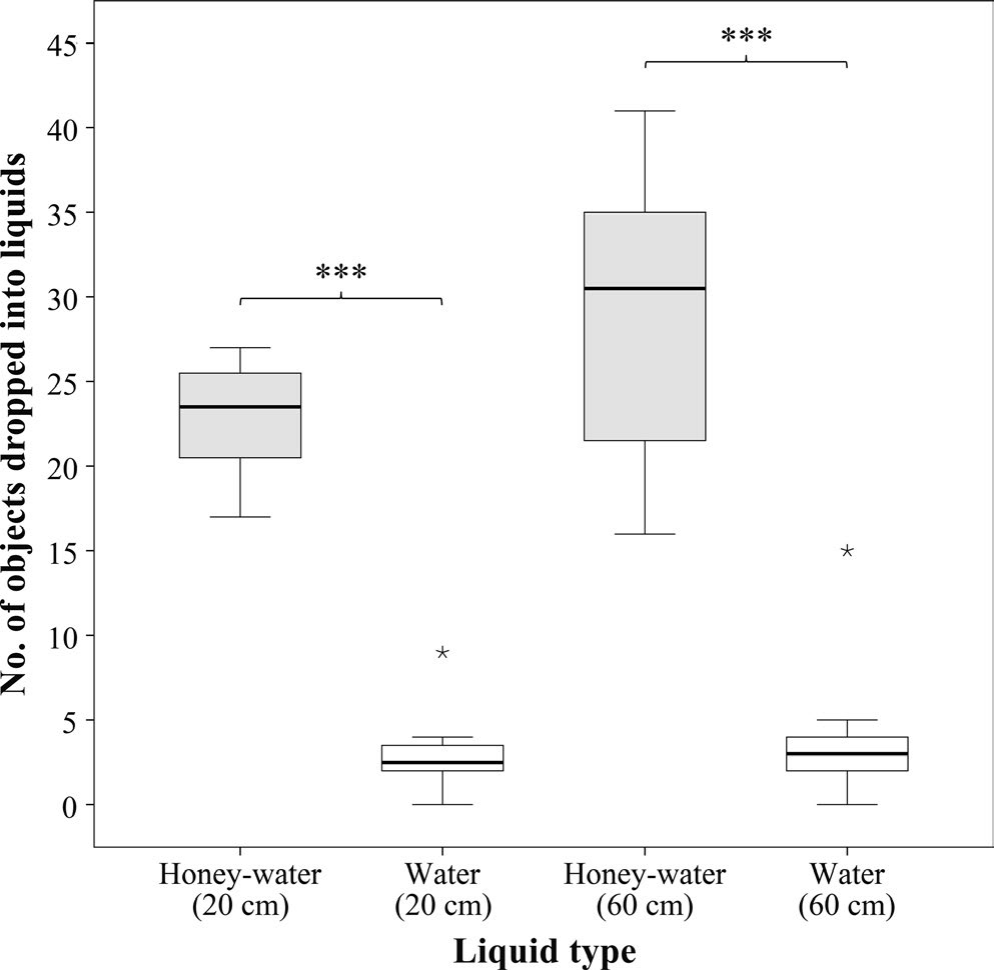
Boxplot showing the number of objects dropped into different types of liquids by the workers of *Aphaenogaster subterranea* when liquids were located either 20 cm or 60 cm from the nests (*N* = 7,200). Significant differences are indicated (***: *p *< .001)

Overall, significantly more objects were dropped (a) into honey‐water than into water (*z* = 13.11, *N* = 7,200, *p* < .001), and (b) into liquids farther (60 cm) from the nest than into those closer (20 cm) to the nest (*z* = 2.10, *N* = 7,200, *p* < .05; Figure 1). In honey‐water, small soil grains were the most (5.69 < *z* < 9.11, *N* = 7,200, *p* < .05) and large soil grains the second most frequently used objects (2.72 < *z* < 4.49, *N* = 7,200, *p* < .05). There were no significant differences among the rest of the objects (−1.93 < *z* < −0.32, *N* = 7,200, 0.30 < *p* < 1.00).

Unlike their behavior at honey‐water, ants were never observed retrieving any objects from water. Overall, significantly more food‐soaked objects were retrieved from liquids placed farther from the nest than from those placed closer to the nest (*z* = 3.66, *N* = 7,200, *p* < .001). Small soil grains and large soil grains were retrieved from honey‐water in a significantly higher number than any other objects (6.70 < *z* < 12.74, *N* = 7,200, *p* < .001), while there were no significant differences among the rest of the objects (−2.10 < *z* < −0.88, *N* = 7,200, 0.22 < *p *< .90).

### 3.2 Experiment 2: previously covered liquids

**Figure 2:**
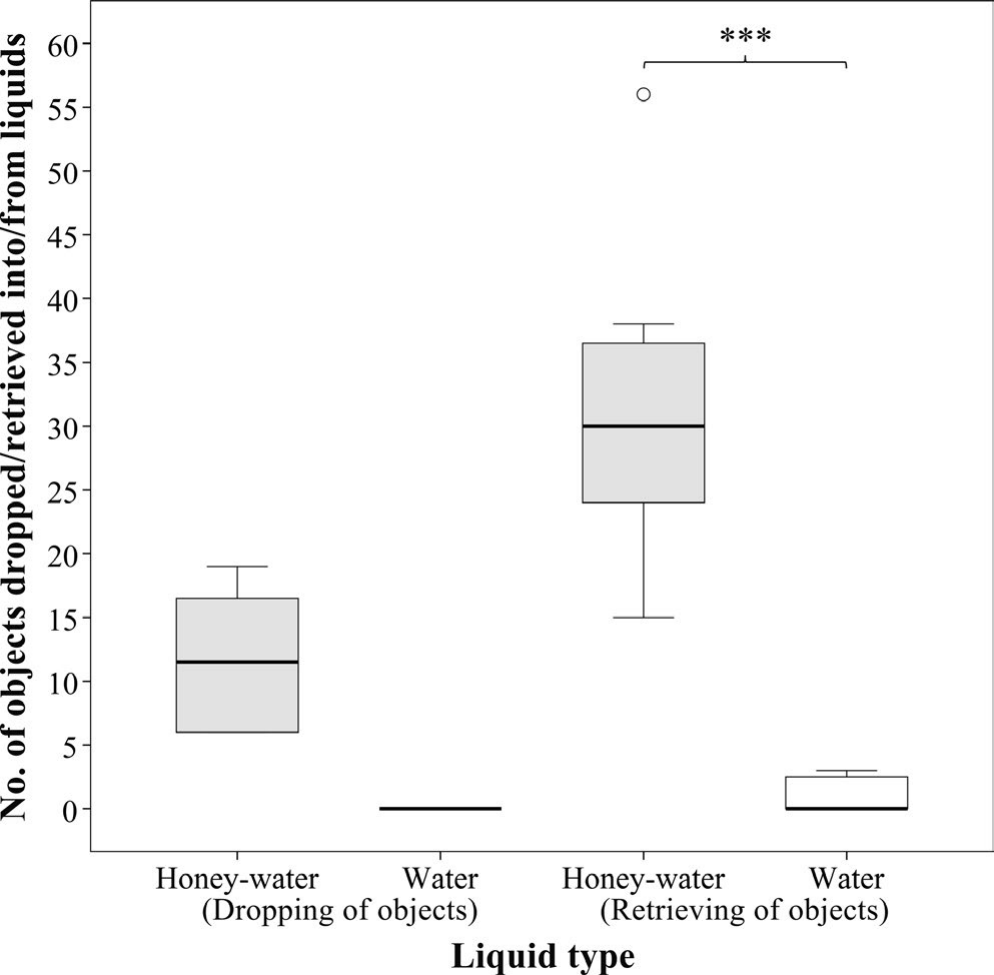
Boxplot showing the number of objects dropped into and retrieved from different types of liquids by the workers of *Aphaenogaster subterranea*when liquids were previously covered with pine needles (*N* = 3,600). Significant differences are indicated (***: *p *< .001)

Unlike their behavior at honey‐water, ants never dropped any additional objects into water (Figure 2). In honey‐water, the least frequently dropped objects were the heaviest, that is, large soil grains and pine needles (−4.13 < *z* < −3.43, *N* = 3,600, *p* < .05), while there were no significant differences among the rest of the objects (0.41 < *z* < 1.40, *N* = 3,600, 0.63 < *p *< .99).

Overall, significantly more objects were retrieved from honey‐water than from water (*z* = 9.53, *N* = 3,600, *p* < .001; Figure 2). Those few objects that were retrieved from water were, however, never transported to the nest, but were scattered in the foraging arena, immediately after they were obtained. In honey‐water, pine needles were the most frequently retrieved objects (4.51 < *z* < 6.70, *N* = 7,200, *p* < .001), while there were no significant differences among the rest of the objects (0.59 < *z* < 3.03, *N* = 7,200, 0.09 < *p *< .98).

### 3.3 Experiment 3: liquid type and size

**Figure 3:**
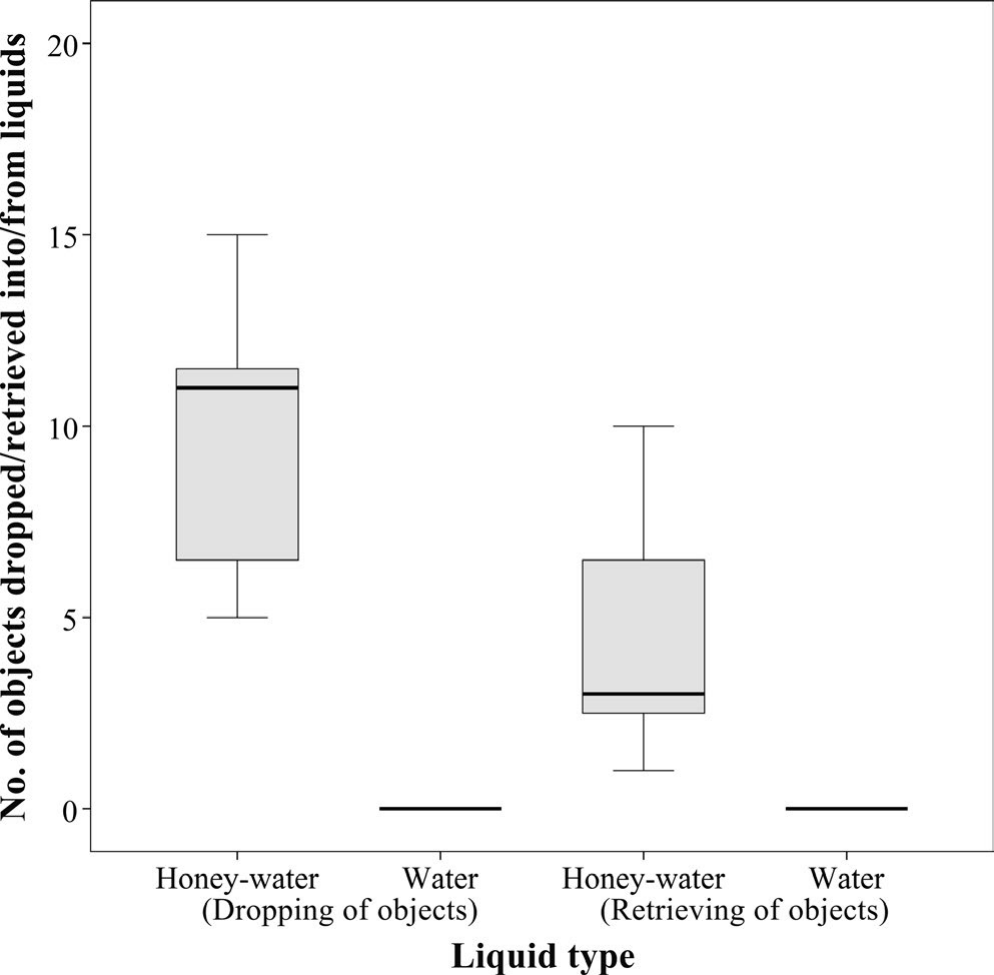
Boxplot showing the number of objects dropped into and retrieved from different types of liquids by the workers of *Aphaenogaster subterranea*when liquids were presented as small droplets (*N* = 3,150)

Unlike their behavior at honey‐water droplets, ants were never observed dropping objects into water droplets (Figure 3). The least frequently dropped objects were large soil grains (−3.64 < *z* < −2.76, *N* = 3,150, *p* < .05), and there was a clear preference toward small soil grains compared with pine needles (*z* = 3.68, *N* = 3,150, *p* < .01). There were no significant differences among the rest of the objects (0.17 < *z* < 2.58, *N* = 3,150, 0.07 < *p* < 1.00).

The most frequently retrieved food‐soaked objects were small soil grains (3.19 < *z* < 3.49, *N* = 3,150, *p* < .05); however, there was no significant difference between small soil grains and pine needles (*z* = 1.42, *N* = 3,150, *p* = .62).

### 3.4 Experiment 4: different nutrition conditions

**Figure 4:**
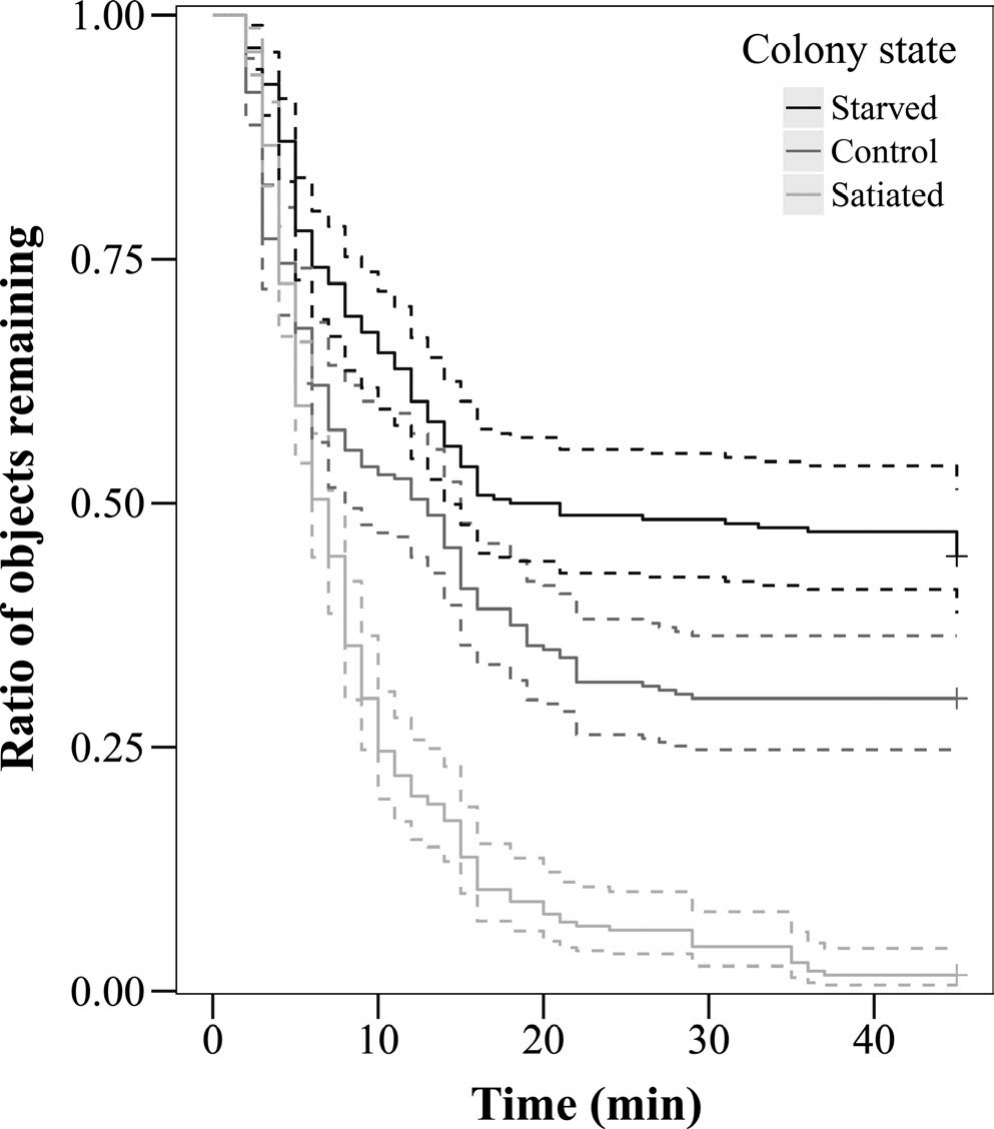
Cox regression showing the ratio of remaining objects (i.e., those not yet dropped into liquids) in starved (black line), control (gray line), and satiated colonies of *Aphaenogaster subterranea* (light gray line) (*N* = 720)

The nutritional condition of colonies had a significant effect on the intensity and rate of the dropping of objects (Figure 4). In satiated colonies, the intensity of object dropping (7.03 < *z* < 10.96, *N* = 720, *p* < .001) and the number of objects dropped were significantly higher (6.73 < *z* < 8.23, *N* = 720, *p* < .001) than in starved and control colonies. Starved colonies showed a lower intensity of object dropping (*z* = −4.25, *N* = 720, *p* < .001), and the number of objects dropped was also significantly lower (*z* = −3.82, *N* = 720, *p* < .001) than in control colonies.

## 4 DISCUSSION

Our study supports that the debris dropping and retrieval behavior of *Aphaenogaster subterranea* constitute "true" tool use (H2). We found, however, no evidence that this behavior represents a two‐component behavior involving two separate processes (i.e., defense response and food transport). Firstly, tool dropping workers made a clear differentiation between honey‐water and water, and they directed more of their foraging efforts toward liquids farther from the nest. Secondly, ants performed tool dropping even into liquids that did not pose a danger of drowning or becoming entangled, that is, honey‐water that was previously covered or was presented as small droplets. Lastly, the nutritional condition of colonies (i.e., starved or satiated) had a significant effect on the intensity and rate of tool dropping, albeit in the opposite direction than we had predicted.

In our study, we observed a clear selectivity between the offered liquid food (honey‐water) and nonfood liquid (water), which is inconsistent with the hypothesis that debris dropping is merely a defensive behavior. Despite that two types of liquids were available for the ants at the same time, significantly more tools were dropped into honey‐water than into water. Our results are corroborated by other studies (Banschbach et al., 2006; Lőrinczi, 2014; Lőrinczi et al., 2018; Maák et al., 2017; McDonald, 1984), which also showed that the debris dropping behavior is selective when it comes to nonfood liquids such as water or petroleum jelly. If foraging workers had indeed considered liquids as a possible threat and they had dropped tools to avoid drowning or entanglement, we would have expected a similar amount of tools dropped into both liquids. Of course, one can assume that stickier liquids like honey‐water could be more threatening and would induce a more intense dropping behavior. This argument is, however, refuted by the finding that viscosity does not have a significant effect on the tool dropping behavior of *A. subterranea* (Lőrinczi et al., 2018). Thus, altogether, the ants seem to drop tools into honey‐water as a response to a discovered food source that can be foraged upon. Therefore, this represents a foraging behavior, not a defensive behavior that arises from a response to a threat posed by the exposed liquid.

According to the second hypothesis (H2), ants dropped significantly more tools into liquids farther from the nest than into those closer to the nest. This finding is consistent with the expectations of central‐place foraging theory (Orians & Pearson, 1979), which predicts that at increasing distances from the nest (the "central place"), an optimal forager will maximize the rate of food delivery in order to offset the costs of traveling. In other words, it is more advantageous to put more effort and/or time in foraging when workers have already travelled a great distance from the nest to a food source. This relation was also confirmed by Davidson (1978), who showed that the workers of *Pogonomyrmex rugosus* had a greater preference for larger seeds than for smaller ones at greater distances from the nest. A similar pattern was found in the study of Schmid‐Hempel (1984), where the workers of *Cataglyphis bicolor* were more persistent in searching a site for food when this site was farther from the nest. Another explanation for our findings may lie in the competitive ability of *Aphaenogaster* species, which usually fell into the subdominant or subordinate group (Cerdá et al., 1997; Fellers, 1987; Holway, 1999; Stukalyuk & Radchenko, 2011). Since food sources farther from the nest are more likely to be found by foragers of other species (including more dominant competitors), their rapid utilization by using tools should be crucial to the foraging success of *A. subterranea*. It is also important to note that dropping tools into food can serve functions other than food transport. Field observations show that other ants are less likely to discover and monopolize food sources that were previously covered with debris by *Aphaenogaster* workers (Banschbach et al., 2006; Fowler, 1982; Lőrinczi, 2014). Even if excluded from the food by dominant competitors, *Aphaenogaster* workers can return at a later time and retrieve the food‐soaked tools ignored by the dominant species, as was observed in *A. rudis* (Fellers & Fellers, 1976).

Contrary to the results of Agbogba (1985), previously covered honey‐water also triggered the tool dropping activity of *A. subterranea*, while water was completely ignored. Two possible explanations could be proposed. One possibility is that ants tried to cover the dangerous surface even more, but again, in this case, we would have expected a similar amount of tools dropped into both liquids. The other possibility is that the intention of further tool dropping was to transport food for the colony. Since there must have been small gaps among pine needles, it is conceivable to assume that workers tried to maximize the number of tools on the food, especially of those that were more suitable for this task. Due to their relatively large size, pine needles can be less easily transported than smaller, more compact tools (e.g., small soil grains, sponges), and transportation speed can play a key role in the foraging of the studied species. Previous studies have shown that the workers of *A. subterranea* optimize tool selection during foraging to minimize foraging time and energy expenditure, therefore prefer small‐sized and easily transportable tools such as small soil grains (Lőrinczi et al., 2018; Maák et al., 2017). This was also evident in this study: workers showed the highest preference for the more manageable tools and the lowest preference for those larger and less manageable ones. Optimization in tool selection may also explain why the mixture of honey and soil particles failed to trigger further tool dropping in *A. subterranea* in Agbogba’s (1985) experiment.

Consistent with the prediction of the tool‐using behavior hypothesis (H2), ants performed tool dropping at liquids presented as small droplets, but only in cases where honey‐water was offered. These droplets were so small that ants could place only one or two tool items over them. Water, just as in the second experiment, was completely ignored. Owing to their small size, droplets were probably not considered as a hazardous surface by workers, so if tool dropping represented a general defense response, we would have experienced only a simple feeding behavior at honey‐water without placing any tools over the droplets. These results, together with our observations that ants continue tool dropping even when there are only barely visible remnants of the liquid food, further illustrate that it is not the threat of drowning or entanglement, but is rather a food‐retrieval response that triggers the dropping behavior in the presence of liquid food. Another important aspect of these results is that they may offer an explanation for the ecological context (i.e., the occurrence and origin) of foraging tool use in ants. Although this behavior was first observed decades ago, to date, no direct evidence has been provided that it is a commonly occurring phenomenon in nature. Fellers and Fellers (1976) noted that the rotting fruit pulp and body fluid of arthropods triggered tool dropping in *Aphaenogaster* species. However, based on the frequency of the occurrence of such food sources, it is still a question whether they played an important role in the evolution of tool‐using behavior. The hemolymph of injured or dead arthropods, for instance, clots very quickly, and after that ants can simply bite pieces out of it (Agbogba, 1985) or just carry the whole carcass back to the nest (Lőrinczi, pers. obs.). Droplets of honeydew released by various hemipterans (e.g., aphids, scales, hoppers), which is a commonly available food source for ants (Delabie, 2001; Hölldobler & Wilson, 1990), however, may explain the occurrence of tool use in nature. We can hypothesize that honeydew fallen to the ground beneath plants could serve as food for *Aphaenogaster* species, and the need to exploit this type of food source may have facilitated the evolution of tool use in the past. Our observations that the ants use tools to carry small droplets of honey‐water seem to support this idea.

In line with the prediction of the tool‐using behavior hypothesis (H2), the nutritional condition of colonies significantly affected the intensity and rate of tool dropping, but in the opposite direction than we expected. Since foraging tool use seems to represent a more efficient way of food transport in *Aphaenogaster* species, its intensity is expected to positively correlate with the increasing food requirement of the colony. Contrary to this prediction, the intensity of tool dropping and the number of tools dropped were significantly higher in satiated colonies than either in starved or control colonies. One possible explanation is simply that, after a long starvation, workers are eager to satisfy their own sugar needs before transporting food back to the nest. Prolonged feeding following colony starvation is well known in other ant species (Josens & Roces, 2000). In such cases, tool dropping workers are hindered by a wall of feeding individuals, requiring them to crawl over the backs of the latter in order to reach the liquid surface. Another possible explanation is that the higher intensity and rate of tool dropping in the satiated state is the result of the above‐mentioned food‐burying behavior that aims to conceal excess food from competitors. This food can then be retrieved sometime in the future when colonies are food deprived. A similar result was obtained in a study with *S. invicta*, in which honey was offered to colonies under different nutritional conditions (Barber et al., 1989). If colonies had been starved, workers ate the honey on the spot, while if colonies had been satiated, workers dropped debris into honey, and within a few hours or days later they transported the food‐soaked tools back to the nest. A recent study also showed that the satiated workers of *S. invicta* display a very similar food‐burying behavior when encountering solid food (Qin et al., 2019). A third possible explanation is that colonies that once experienced prolonged starvation compensate by utilizing food in greater amounts in order to be prepared for another starvation period. A similar phenomenon was described for *Formica polyctena* foraging on nestmate corpses after a satiation period following feeding stress (Maák et al., 2020). In satiated colonies, the majority of corpses that were offered them were taken back to the nest and consumed in greater quantities than in control (before starvation) colonies. It is important to note, however, that despite the difficulty of interpreting these results, our findings argue against the two‐component behavior hypothesis (H1). If tool dropping represented a general defense response against drowning or entanglement, we would have observed a similar intensity and rate of tool dropping irrespective of the nutritional condition of colonies.

Although our results do not preclude the possibility that debris dropping functions primarily to conceal excess food from competitors, some evidence indicates that the main purpose of this behavior is rather to promote food transport, at least in our study species, *A. subterranea*. Based on an unpublished study (Módra et al., in prep.), *A. subterranea* colonies with normal nutrition condition (being fed every second day) can completely retrieve honey‐water by using tools within just a few hours. Moreover, tool dropping and retrieving are simultaneous processes in this species, making the foraging really effective, because when uncovered liquid surfaces are exposed, ants place new tools instead of the retrieved ones. Furthermore, a recent study shows that tool use in *A. subterranea* exhibits a high degree of flexibility; that is, tool selection varies as a function of liquid type, distance, and availability of tools, and also as a function of whether ants are dropping tools into or retrieving tools from liquids. For instance, workers use larger tools when trying to cover nonfood liquid surfaces, while they show strong preferences for smaller and easily transportable tools in liquid foods (Lőrinczi et al., 2018). In addition, ants are able to learn how to improve the use of certain tools (such as sponges) by modifying them, thereby facilitating the handling of these tools during foraging. It is a question, however, whether ants react differently when trying to cover nonfood liquid surfaces (as a general defense response) or a food source that needs to be concealed from competitors. It may also be that debris dropping has a dual function depending on the foraging environment: to bury and preserve food in some instances, while in other cases to transport food immediately back to the nest. For clarification of this issue, special experiments are needed with a larger amount of food and with the presence of competitors, and also longer observations and repeated alternations of starvation and satiation periods.

In conclusion, our results, together with those of previous studies on the foraging behavior of *Aphaenogaster* species, suggests that the debris dropping and retrieving behavior of the members of this genus are more complex than that predicted by the two‐component behavior hypothesis and deserves to be considered as "true" tool use. Moreover, there are no such criteria in the current definition of tool use which would exclude this behavior because of the time gap between the dropping and retrieving of tools (Bentley‐Condit & Smith, 2010). We are, however, ready to accept that in closely related genera such as *Messor*, debris dropping represents either the ancestral protective behavior from which tool use has evolved in *Aphaenogaster* species (Fellers & Fellers, 1976), or, if we suppose that tool use was already present in the last common ancestor of the two genera, a simplification in the behavior that may be attributed to the shift in foraging strategy (i.e., feeding largely on seeds). In fact, our comparative studies with *A. subterranea* and *Messor structor* show that there are substantial differences in the tool dropping and retrieving behavior of the two species both in intensity and effectiveness (Módra et al. in prep.). It should be also pointed out that the evolution of this behavior is highly linked to the cognitive capabilities of social insects, whose limits are still widely unknown.

## CONFLICT OF INTEREST

We have no conflicts of interest to report for this manuscript.

## AUTHOR CONTRIBUTION

**Gábor Módra:** Conceptualization (equal); Data curation (equal); Investigation (equal); Methodology (equal); Visualization (equal); Writing‐original draft (equal); Writing‐review & editing (equal). **István Maák:** Conceptualization (equal); Investigation (equal); Methodology (equal); Software (lead); Writing‐review & editing (equal). **Ádám Lőrincz:** Investigation (equal); Methodology (equal); Writing‐review & editing (equal). **Orsolya Juhász:** Investigation (equal); Methodology (supporting); Writing‐review & editing (supporting). **Péter János Kiss:** Investigation (equal); Methodology (supporting); Writing‐review & editing (supporting). **Gábor Lőrinczi:** Conceptualization (equal); Data curation (equal); Investigation (equal); Methodology (equal); Visualization (equal); Writing‐original draft (equal); Writing‐review & editing (equal).

## Data Availability

The data underlying all the results presented in the paper is available from the Dryad Digital Repository: https://doi.org/10.5061/dryad.1zcrjdfqp
